# Comparison of the geometry of the left ventricle outflow tract, the aortic root and the ascending aorta in patients with severe tricuspid aortic stenosis versus healthy controls

**DOI:** 10.1007/s10554-019-01715-5

**Published:** 2019-11-04

**Authors:** Małgorzata Nieznańska, Karina Zatorska, Patrycjusz Stokłosa, Małgorzata Ryś, Piotr Duchnowski, Piotr Szymański, Tomasz Hryniewiecki, Ilona Michałowska

**Affiliations:** 1 Department of Acquired Cardiac Defects, Institute of Cardiology, Alpejska 42, Warsaw, Poland; 2 Department of Radiology, Institute of Cardiology, Ul. Alpejska 42, Warsaw, Poland

**Keywords:** Aortic root, Aortic stenosis, Multislice computed tomography

## Abstract

The purpose of this study was to assess by multislice computed tomography (MSCT) imaging geometry of the ascending aorta, the aortic root, the aortic annulus and the left ventricle outflow tract (LVOT) in aortic stenosis (AS) patients, to compare aortic root morphology in patients with AS with healthy controls and to evaluate sex differences. Fifty patients with severe AS and 50 age- and gender-matched controls who underwent MSCT were included in the study. The dimensions of the LVOT, the aortic annulus, the aortic root, the ascending aorta, and the volume of the aortic root were retrospectively assessed and a comparison was made between patients with severe tricuspid AS and controls. Patients with tricuspid AS in comparison with controls had smaller dimensions of the sinus of Valsalva resulting in reduction of the aortic root volume, whereas the dimensions of the other structures were comparable. MSCT revealed larger annular, LVOT and the sinus of Valsalva dimensions and the aortic root volume in men than women. Men with AS differed from healthy men only in regard to the dimensions of the sinus of Valsalva, while women showed significant differences also in the LVOT, and the aortic annulus. MSCT showed accurately aortic root remodeling in tricuspid AS patients and indentified sex-dependent differences. Women with tricuspid AS differ from healthy women more than men did. A high degree of the variability in the aortic root dimensions requires further careful research.

## Introduction

Aortic stenosis (AS) is the most common native valve disease [1] occurring more and more frequently due to the ageing population [2]. Aortic valve is a part of the sophisticated structure of the aortic root. The aortic root is the continuation of the left ventricular outflow tract (LVOT), which is located between the basal attachment of the aortic valvar leaflets and the sinotubular junction (STJ) [3]. It includes the aortic valve annulus with leaflets and the sinus of Valsalva [3].

Detailed knowledge of the aortic root geometry is essential to understand the mechanism or pathophysiology of valvular heart disease and clinical consequences resulting from valvular disruption. Careful assessment of the aortic root geometry is crucial to surgical repair techniques used for treatment various diseases of the aortic root. Moreover, in the era of transcatheter aortic valve implantation (TAVI), advanced and precise evaluation is essential in the accurate positioning of the prosthesis in the aortic annulus, prosthesis sizing as well as avoidance of the covering of the coronary ostia by the upper part of the prosthesis or occlusion of the left coronary artery [4]. Currently there is still paucity of data about detailed assessment of the aortic root geometry in aortic stenosis compared to both healthy people and between genders and the further studies are necessary.

The preferred imaging tool to assess the anatomy and dimensions of the aortic root is multislice computed tomography (MSCT) [2].

The aim of this study was to characterize geometry of the ascending aorta, the aortic root, the aortic annulus and the left ventricle outflow tract (LVOT) in aortic stenosis, to assess whether aortic root geometry differs between AS patients and those without valvular heart disease, and to identify sex differences.

## Materials and methods

### Study group

Retrospectively, aortic root geometry was assessed and compared between patients with severe tricuspid aortic valve stenosis and controls without valvular disease in this single center study. All data were indexed to the body surface area (BSA).

25 consecutive women and 25 consecutive men with severe AS who underwent MSCT and transthoracic echocardiography (TTE) before TAVI in 2015-2018 at our Institute were included in the study. Age-matched 25 women and men without valvular pathology, who were subjected to coronary angiography CT due to suspected coronary artery regression were enrolled in a control group.

Exclusion criteria included renal failure, hypersensitivity to iodinated contrast material and bicuspid aortic valve. The aspect of the aortic valve, the tricuspid or the bicuspid one were assessed by TTE and MSCT.

### Multislice computed tomography

All patients underwent computed tomography scanning using dual source scanner Somatom Force (Siemens Healthcare, Forchheim, Germany) with the following parameters: 2 × 192 slices with 0.6-mm collimation, gantry rotation time 250 ms, tube voltage 70-100 kV, tube current 320–500 mAs (depending on the patient body mass). In both groups, non-enhanced prospectively ECG-gated scan (75% of R–R interval) with a slice thickness of 3 mm was performed to measure the calcium score of the aortic valve according to the modified Agatston method using dedicated calcium scoring software [5].

After a non-enhanced scan, a retrospectively ECG-gated helical CT angiography was performed in both groups (controls and in patients with severe AS). To minimize radiation exposure, electrocardiographically-gated tube current modulation was applied in all patients. The scan covered the heart (extending from the tracheal bifurcation to the diaphragm). In patients with AS, the ECG-gated data acquisition was followed by a non–ECG-gated CT angiographic scan of the chest, the abdomen, and the pelvis for the assessment of an access route. Iodinated contrast material (350–370 mg/ml) was injected intravenously at a flow rate of 4.5 ml/s.

CT acquisition parameters were slice collimation 192 × 0.6 mm, gantry rotation time 250 ms, tube voltage 70–100 kV, tube current 320–500 mAs (depending on the patient body mass), pitch of 0.16–0.3 (depending of the heart rate).

The dataset of the contrast-enhanced scan was reconstructed every 10% of the R–R interval.

Detailed assessments of aortic root anatomy and the ascending aorta were performed. Measurements were taken using dedicated software syngo.via (Siemens Healthcare, Forchheim, Germany).

The aortic annulus was assessed in the oblique transversal plane which crossed the level of the most basal attachments of the aortic cusps. The dimensions of the aortic root at the level of the STJ and the widest portion of the sinus of Valsalva were measured in the oblique transversal plane perpendicular to the course of the aorta [6].

**Fig. 1:**
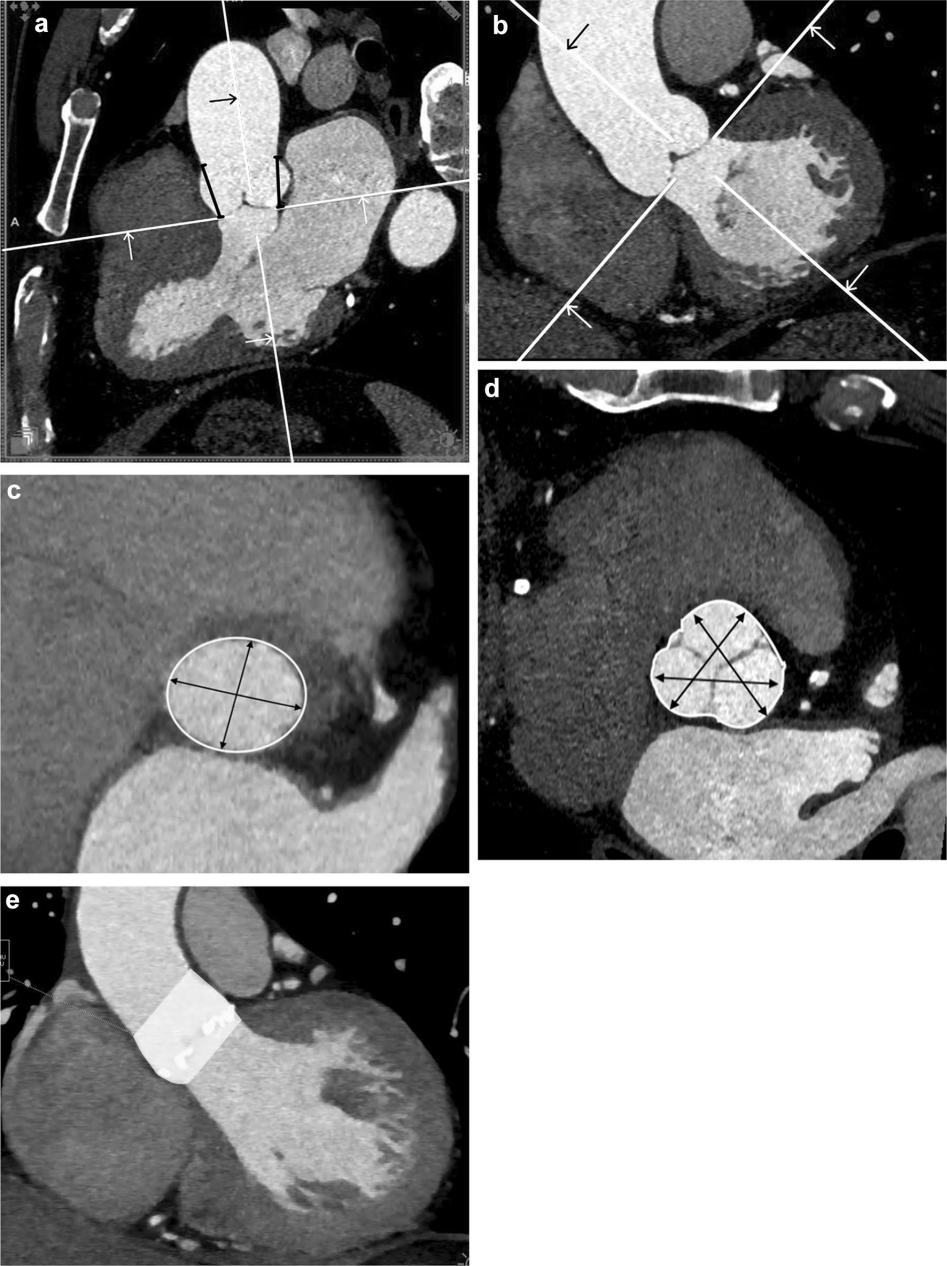
The measurements of the aortic root on the multislice computed tomography. **a** The coronal oblique plane through the long axis of the left ventricular outflow tract. Measurements of the height at the left and the right side of the sinus of Valsalva- black lines. **b** The sagittal oblique plane view through the long axis of the left ventricular outflow tract. **c** Short-axis view of the aortic annulus. The aortic annulus is measurement just below the lowest insertion points of the aortic leaflets. The black arrows show the long- and short-axis diameters and the white line shows the annular circumference and area. **d** Short-axis view of the sinus of Valsalva (the plane parallel to the annular plane) on the level of widest dimensions. The black arrows show diameters from one sinus to another sinus and white line shows the sinus circumference and Fig. 2. Box plots comparing dimension of the sinus of Valsalva between AS patients and control group showing mean value, median, standard deviation, quantiles and outliers

The following measurements of the aortic root and the aortic valve were performed using MPRs: the long axis (maximum) of annulus, the short axis (minimum) of the annulus, the area of annulus (defined as an oval or a circle formed by linking the most basal portions of the leaflet attachments) [6], the perimeter of the annulus, the widest and the highest portion of the sinus of Valsalva diameter, the area and the perimeter of the sinus of Valsalva (Fig. 1a–d). The sinus of Valsalva was measured on the level of the widest diameter from one sinus to another sinus. The average of three sinus-to-sinus measurements were calculated. The sinus of Valsalva height was measure from the annular plain to the highest point of the right and the left sinus of Valsalva. The area, the perimeter and the diameter of the ascending aorta were measured 4.5 cm above the annulus on a transverse double oblique plane perpendicular to the long axis of the ascending aorta. The volume of the aortic root was calculated from the annular plane to the lowest point of the sinotubular junction using syngo.via VOI freehand Siemens software (Fig. 1e). All diameter measurements were assessed in the systolic phase. The aortic wall and calcifications were included to all dimensions.

The shape of the LVOT, the aortic annulus, the STJ and the ascending aorta was assessed by the ellipticity index (EI). The ellipticity index defined as maximal diameter (Dmax) divided by minimal diameter (Dmin) and the circular shape was established for EI < 1.1, which means that the difference between Dmax and Dmin was <10% of Dmin [4].

All analyses were assessed by a single expert reader experienced in cardiac CT and trained in the interpretation of CT scans before TAVI procedure.

### Echocardiography

A standard comprehensive TTE was performed in each patient. The Vivid S70 and E9 (General Electric Medical Systems, Milwaukee, WI, USA) were used. Echocardiographic measurements were performed by qualified echocardiographers experienced in the quantitative assessment of valvular heart diseases.

According to the latest guidelines of the European Society of Cardiology (ESC) [2], severe AS was determined quantitatively on the basis of the aortic valve area < 1.0 cm^2^, mean aortic gradient > 40 mmHg, or aortic jet velocity > 4.0 m/s.

TTE data were collected, including the left ventricular end-diastolic diameter (LVEDD), the LV end-systolic diameter (LVESD), the interventricular septum (IVS) diameter at the end-diastole, and the end-diastolic posterior wall thickness (PW), the left ventricle ejection fraction (LVEF). All measurements were made in the parasternal long axis view.

### Statistical analysis

All data were presented as absolute values and values indexed to BSA.

Continuous data were presented depending on the distribution as mean ± standard deviation (SD) or median and range of values, and categorical variables are presented as frequency and percentages. The data distribution was verified by the Kolmogorov–Smirnov test. The T-test, the Chi square, and the Mann–Whitney test were performed for data analysis. Correlations between variables were assessed with the Pearson’s correlation coefficient. All P values were two-sided and a P value < 0.05 was considered to be statistically significant.

### Ethics statement

The study was carried out in accordance with the Declaration of Helsinki of the World Medical Association. The protocol for the study was reviewed and approved by the Ethics Committee on Human Studies (IK-NPIA-0021-74/1733/2018). Each participant read and signed the informed consent form after study procedures had been fully explained. Study subjects were not compensated for their participation. This article does not contain any studies with animals performed by any of the authors.

## Results

The baseline clinical characteristics and echocardiographics data of the study and the control group are summarized in Table 1.

**Table 1:** Demographic, clinical, laboratory and echocardiographics characteristics of patients

	Study group (n = 50)	Control group (n = 50)	p value
Age (years)	74.4 ± 6.9	74.4 ± 5.7	0.99
Male	25 (50%)	25 (50%)	1
Weight (kg)	80.6 ± 16.7	80 ± 12	0.85
Height (cm)	166 ± 8.7	170 ± 11.8	0.59
Body mass index (kg/m^2^)	29.5 ± 7	27.7 ± 3.3	0.11
Body surface area (m^2^)	1.9 ± 0.2	1.9 ± 0.2	0.75
Past medical history			
Hypertension	36 (72%)	36 (72%)	1
Coronary artery disease	33 (66%)	35 (70%)	0.67
Atrial fibrillation	14 (28%)	16 (32%)	0.66
Diabetes mellitus	22 (44%)	8 (16%)	0.02
eGFR (ml/min/1.63 m^2^)	64.3 ± 20.9	67.3 ± 15	0.44
Echocardiographics data			
LV end-systolic diameter, (mm)	30.3 ± 7.2	32.1 ± 10.2	0.46
LV end-diastolic diameter, (mm)	48.5 ± 7.3	49.8 ± 7.3	0.4
IVS in diastole, (mm)	14.9 ± 2.4	11.7 ± 2.1	<0.001
PW in diastole, (mm)	11.8 ± 2	10.5 ± 1.9	0.005
LVEF, (%)	57.1 ± 12.9	56.1 ± 11.6	0.73
Left atrial diameter (cm)	4.3 ± 1.3	4.3 ± 0.7	0.99
Left atrial area (cm^2^)	25.5 ± 4.8	24.2 ± 7.1	0.42
Severe mitral regurgitation	2 (4%)	0 (0%)	0.31
Severe aortic regurgitation	1 (2%)	0 (0%)	0.92
Severe tricuspid regurgitation	1 (2%)	0 (0%)	0.92
Aortic valve area (equation of continuity), (cm^2^)	0.6 ± 0.2		
Peak aortic gradient, (mm Hg)	85.1 ± 28.2		
Mean aortic gradient, (mmHg)	52.6 ± 16.8		
Maximal aortic valve velocity (m/s)	4.6 ± 0.7		

Absolute aortic valve calcium score and indexed to body surface area were higher in patients with AS compared with controls (3050.45 ± 1412.3 AU vs. 26.09 ± 73.9 AU; p < 0.001 and 1619.29 ± 783.1 AU/m^2^ vs. 12.36 ± 35.1 AU/m^2^; p < 0.001 respectively). Differences in calcium score between men and women with AS were not found (3139.8 ± 1494.9 AU vs. 2961.1 ± 1349.5 AU; p = 0.7 respectively).

**Fig. 2:**
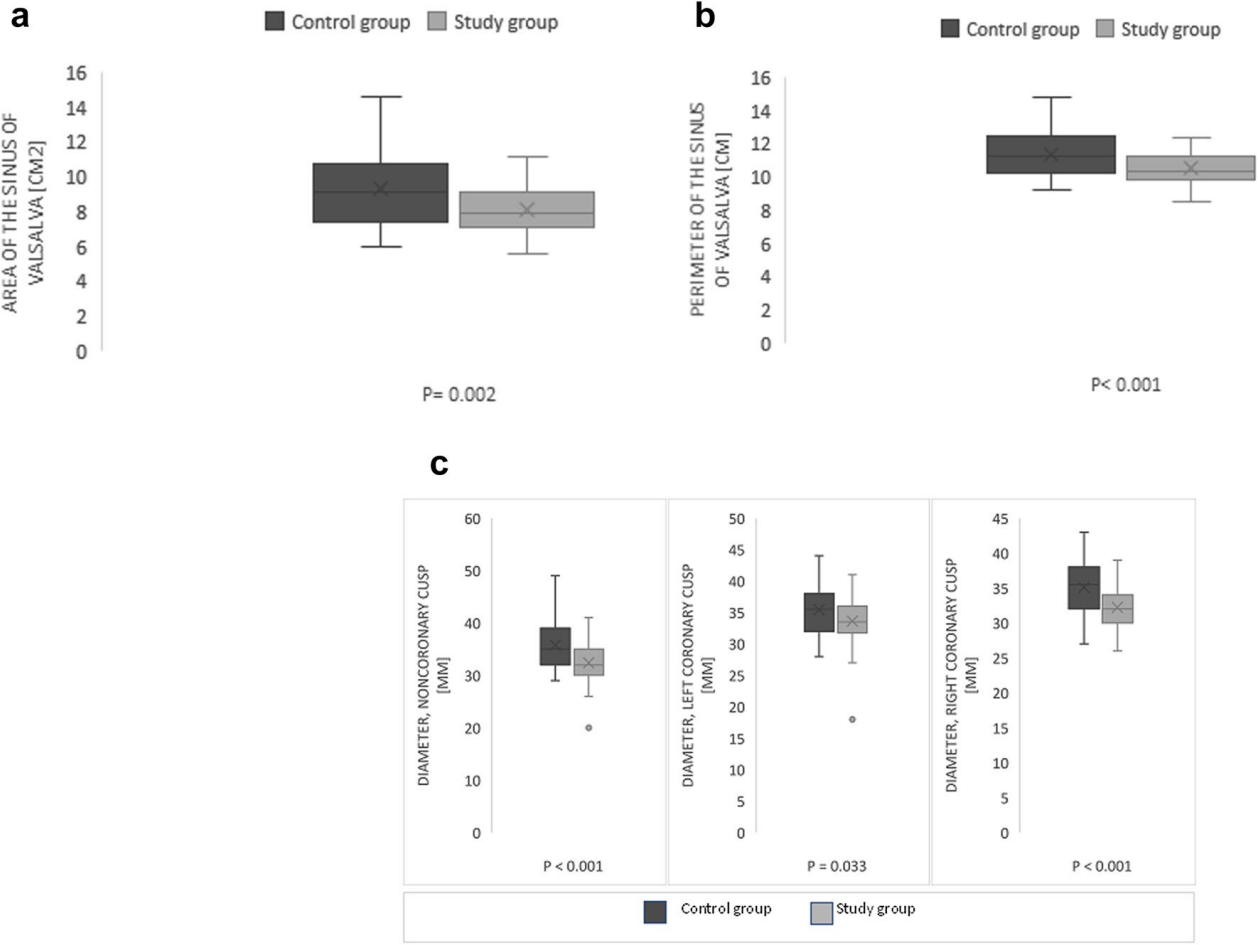
**a** Patients with AS compared with healthy controls have smaller area of the sinus of Valsalva (p < 0.001). **b**. Patients with AS compared with patients without AS have smaller perimeter of the sinus of Valsalva (p < 0.001). **c** Patients with AS got smaller sinus of Valsalva with regard to all diameters-noncoronary cusp (p < 0.001), left coronary cusp (p = 0.033) and left coronary cusp (p < 0.001)

**Fig. 3:**
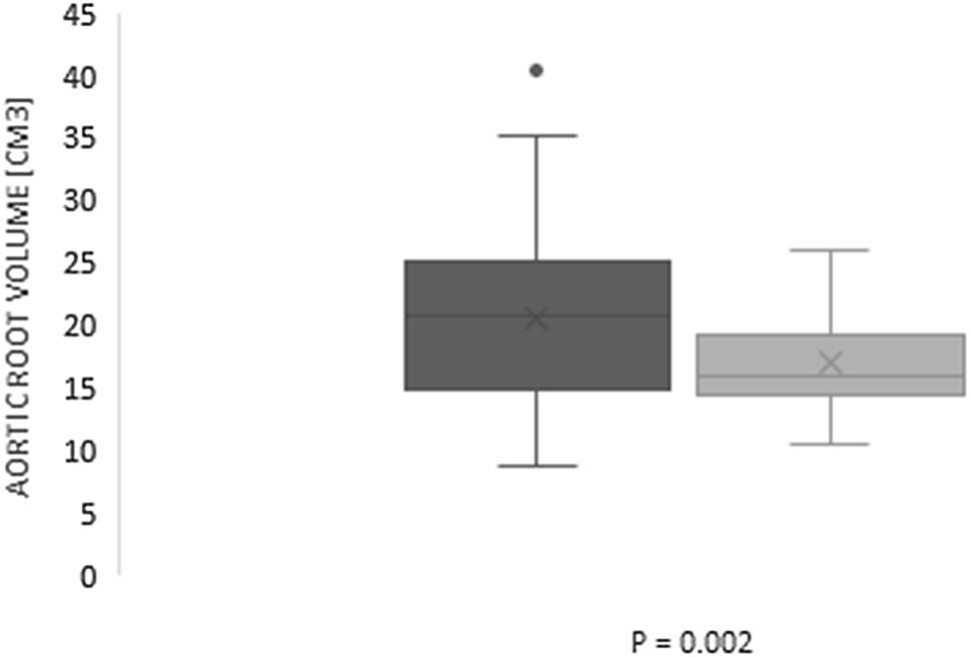
Box plots comparing volume of the aortic root between AS patients and control group. Patients with AS got reduced volume of the aortic root compared to patients without AS

Comparison of the aortic annulus, the LVOT, the sinus of Valsalva, the STJ and the ascending aorta between the two groups were shown in Table 2 and Fig 2. The average aortic root volume was 17 ± 3.9 cm^3^ in the study group and 20.6 ± 6.7 cm^3^ in the control group (p = 0.002) (Fig. 3).

**Table 2:** Comparison of the aortic annulus, the LVOT, the sinus of Valsalva, the STJ and the ascending aorta dimensions between study and control group

	Absolute value			Indexed to BSA		
	Study group (n = 50)	Control group (n = 50)	p value	Study group (n = 50)	Control group (n = 50)	p value
Aortic annulus						
Area (cm^2^)	4.6 ± 0.7	4.5 ± 1	0.64	2.4 ± 0.4	2.3 ± 0.5	0.33
Perimeter (mm)	107 ± 143	76 ± 10	0.14	55 ± 6.9	40 ± 5	0.14
Diameter max (mm)	27.8 ± 2.4	27.2 ± 3.1	0.24	14.7 ± 2	14.1 ± 1.4	0.11
Diameter min (mm)	21.6 ± 2	21.4 ± 2.5	0.64	11.4 ± 1.5	11.1 ± 1.1	0.34
Left ventricular outflow tract						
Area (cm^2^)	4.6 ± 1.1	4.4 ± 1.2	0.43	2.4 ± 0.6	2.2 ± 1.06	0.43
Perimeter (mm)	81 ± 9	79 ± 9	0.31	43 ± 7	41 ± 4	0.15
Diameter max (mm)	29.5 ± 3.3	29.3 ± 3.2	0.37	15.6 ± 2.6	14.9 ± 2.4	0.2
Diameter min (mm)	19.8 ± 2.8	19.1 ± 3.4	0.29	10.4 ± 1.8	9.9 ± 1.5	0.13
Sinus of Valsalva						
Area (cm^2^)	8.2 ± 1	9.3 ± 2	0.002	4.3 ± 1	4.8 ± 1	0.14
Perimeter (mm)	105 ± 10	114 ± 10	< 0.001	55 ± 10	59 ± 10	0.06
Diameter, noncoronary cusp (mm)	32.4 ± 4	35.8 ± 5	< 0.001	17.1 ± 3	18.6 ± 3	0.02
Diameter, left coronary cusp (mm)	33.6 ± 4	35.4 ± 4	0.033	17.7 ± 3	18.4 ± 2	0.14
Diameter, right coronary cusp (mm)	32.2 ± 3	35.1 ± 4	< 0.001	17 ± 2	18.2 ± 2	0.02
Height, left side (mm)	19.8 ± 3	20.5 ± 2	0.12	10.4 ± 2	10.7 ± 1	0.31
Height, right side (mm)	19.7 ± 3	21.1 ± 3	0.02	10.4 ± 2	11 ± 2	0.08
Sino-tubular junction						
Area (cm^2^)	6.5 ± 1.3	6.9 ± 1.8	0.052	3.4 ± 0.8	3.6 ± 0.9	0.08
Perimeter (mm)	88.6 ± 11.7	93.3 ± 16.8	0.008	46.7 ± 7.7	48.5 ± 8.5	0.04
Diameter max (mm)	29 ± 3.1	30 ± 3.3	0.14	15.2 ± 2.2	15.4 ± 1.5	0.56
Diameter min (mm)	27.9 ± 2.9	29.2 ± 3.3	0.04	14.8 ± 2	15.3 ± 1.6	0.12
Ascending aorta						
Area (cm^2^)	9.2 ± 2	9 ± 2.1	0.75	4.8 ± 1.1	4.7 ± 1.1	0.89
Perimeter (mm)	107.6 ± 10.9	105.7 ± 18.1	0.71	56.7 ± 7.8	55 ± 9.9	0.81
Diameter max (mm)	34.5 ± 3.4	34.4 ± 3.5	0.95	18 ± 2.3	17.8 ± 1.9	0.69
Diameter min (mm)	33.5 ± 3.5	33.8 ± 3.5	0.73	17.8 ± 2.4	17.7 ± 2	0.81

The aortic annulus was oval (EI > 1.1) in 98% (n = 49 out of 50) of the study group and in 98% (n = 49 out of 50) of the controls. No circular shape of LVOT was observed in both groups. Overall, the ascending aorta and the STJ were circular in most patients in both groups, with oval shapes in 4% (n = 2 out of 50) and 16% (n = 8 out of 50) in the study group and 2% (n = 1 out of 50) and 0% (n = 0 out of 50) in the control group, respectively.

Sex-dependent dimensions of the aortic root were shown in Table 3.

**Table 3:** Sex-specific dimensions of the aortic root in patients with AS

	Absolute value			BSA indexed		
	Male (n = 25)	Female (n = 25)	p value	Male (n = 25)	Female (n = 25)	p value
Aortic annulus						
Area (cm^2^)	5 ± 0.7	4.2 ± 0.5	< 0.001	2.6 ± 0.4	2.3 ± 0.4	0.03
Perimeter (mm)	111 ± 146	103 ± 143	0.86	56.7 ± 73	53.5 ± 65	0.87
Diameter max	28.9 ± 3	26.8 ± 2	0.001	14.9 ± 2	14.4 ± 2	0.44
Diameter min	22.4 ± 2	20.7 ± 1.4	0.001	11.6 ± 1.6	11.2 ± 1.4	0.39
Left ventricular outflow tract						
Area (cm^2^)	5.1 ± 1.3	4.2 ± 0.5	0.002	2.6 ± 0.8	2.3 ± 0.4	0.03
Perimeter (mm)	85.4 ± 10.3	77.4 ± 5.3	0.001	44.1 ± 7.5	41.8 ± 5.7	0.23
Diameter max	30.4 ± 3.9	28.6 ± 2.4	0.05	15.7 ± 2.9	15.4 ± 2.2	0.67
Diameter min	20.6 ± 3.4	18.9 ± 1.6	0.02	10.6 ± 2.1	10.2 ± 1.4	0.36
Sinuses of Valsalva						
Area (cm^2^)	9.1 ± 1.3	7.3 ± 1	< 0.001	4.7 ± 0.8	3.9 ± 0.6	0.001
Perimeter (mm)	111 ± 8.5	99.2 ± 6.8	< 0.001	57.1 ± 7.3	53.5 ± 6.5	0.07
Diameter, noncoronary cusp (mm)	33.9 ± 4.2	30.9 ± 2.5	0.003	17.5 ± 2.8	16.6 ± 2	0.23
Diameter, left coronary cusp	35.2 ± 4.6	32 ± 2.6	0.004	18.1 ± 3	17.3 ± 2.3	0.25
Diameter, right coronary cusp	33.6 ± 2.9	30.8 ± 2.2	< 0.001	17.3 ± 2.2	16.6 ± 2.1	0.27
Height, left side (mm)	20.9 ± 2.9	18.6 ± 1.3	0.001	10.7 ± 1.7	10.1 ± 1.4	0.13
Height, right side (mm)	20.8 ± 3.1	18.7 ± 2.2	0.01	10.7 ± 1.8	10.1 ± 1.7	0.26
Aortic root volume (cm^3^)	19.1 ± 3.7	14.9 ± 2.9	< 0.001	9.9 ± 2.2	8 ± 1.6	0.001
Sino-tubular junction						
Area (cm^2^)	7 ± 1.5	6 ± 1	0.02	3.6 ± 0.9	3.2 ± 0.6	0.22
Perimeter (mm)	89.5 ± 15	87.7 ± 6.8	0.22	46.2 ± 9.1	47.2 ± 5.9	0.54
Diameter max	29.9 ± 3.4	28.1 ± 2.4	0.054	15.5 ± 2.6	15 ± 1.8	0.44
Diameter min	28.8 ± 3.1	27 ± 2.4	0.03	14.8 ± 2.2	14.7 ± 1.7	0.81
Ascending aorta						
Area (cm^2^)	9.3 ± 2.3	9 ± 1.6	0.9	4.8 ± 1.2	4.8 ± 0.9	0.43
Perimeter (mm)	108.2 ± 12.5	106.9 ± 8.9	0.83	55.7 ± 8.2	57.6 ± 7.2	0.23
Diameter max	34.9 ± 3.7	34 ± 3.1	0.63	17.9 ± 2.6	18.1 ± 2	0.77
Diameter min	34 ± 3.9	33.1 ± 3	0.59	17.6 ± 2.5	18 ± 2.3	0.53

AS men compared with men without AS got smaller sinus of Valsalva with regard to all diameters (33.9 ± 4.2 mm vs. 38.4 ± 3.9 mm; p < 0.001; 35.2 ± 4.6 mm vs. 38.3 ± 3.1 mm; p = 0.008; 33.6 ± 2.9 mm vs. 38.1 ± 2.5 mm; p < 0.001), area (9.1 ± 1.3 cm^2^ vs. 10.7 ± 1.6 cm^2^; p < 0.001) and the perimeter (111.1 ± 0.8 mm vs. 123.1 ± 1.1 mm; p < 0.001). Also aortic root volume was smaller in study group (19.1 ± 3.7 cm^3^ vs. 25.4 ± 5.3 cm^3^; p<0.001). AS men compared with men without AS got smaller the STJ dimensions with regard to the area, the perimeter and the long axis. There were no significant differences in LVOT, aortic annulus and ascending aorta dimensions (Table 4).

**Table 4:** Comparison of the aortic annulus, the LVOT, the sinus of Valsalva, the STJ and the ascending aorta dimensions between male with AS and male without AS and between female with AS and female without AS

	Male AS (n = 25)	Male without AS (n = 25)	p value	Female AS (n = 25)	Female without AS (n = 25)	p value
Aortic annulus						
Area (cm^2^)	5 ± 0.7	5.1 ± 0.8	0.67	4.2 ± 0.5	3.9 ± 0.9	0.17
Perimeter (mm)	111 ± 146	83 ± 7	0.34	103 ± 143	70 ± 9	0.25
Diameter max (mm)	28.9 ± 2.5	29.3 ± 2.7	0.6	26.8 ± 1.7	25.1 ± 1.9	0.002
Diameter min (mm)	22.4 ± 2.1	23 ± 1.9	0.36	20.7 ± 1.4	19.8 ± 1,9	0.05
Left ventricular outflow tract						
Area (cm^2^)	5.1 ± 1.3	5.2 ± 1	0.7	4.2 ± 0.5	3.7 ± 0.7	0.005
Perimeter (mm)	85 ± 10	86 ± 8	0.87	77 ± 5	73 ± 6	0.015
Diameter max (mm)	30.4 ± 3.9	31.4 ± 2.5	0.96	28.6 ± 2.5	27.2 ± 2.2	0.035
Diameter min (mm)	20.6 ± 3.4	21.1 ± 2.9	0.6	18.9 ± 1.6	17.1 ± 2.5	0.004
Sinuses of Valsalva						
Area (cm^2^)	9.1 ± 1,3	10.7 ± 1.6	< 0.001	7.3 ± 1	7.9 ± 1.4	0.022
Perimeter (mm)	111.1 ± 0.8	123.1 ± 1.1	< 0.001	99.3 ± 6.8	104.6 ± 9.5	0.02
Diameter, noncoronary cusp (mm)	33.9 ± 4.2	38.4 ± 3.9	< 0.001	30.9 ± 2.5	33.1 ± 3.7	0.018
Diameter, left coronary cusp (mm)	35.2 ± 4.6	38.3 ± 3.1	0.008	32 ± 2.6	32.5 ± 3	0.55
Diameter, right coronary cusp (mm)	33.6 ± 2.9	38.1 ± 2.5	< 0.001	30.8 ± 2.2	32.1 ± 2.9	0.094
Height, left side (mm)	20.9 ± 2.9	20.9 ± 2.9	0.98	18.6 ± 1.3	20.2 ± 1.6	0.001
Height, right side (mm)	20.8 ± 3.1	22.3 ± 3.1	0.083	18.7 ± 2.2	19.8 ± 2.2	0.08
Aortic root volume (cm^3^)	19.1 ± 3.7	25.4 ± 5.3	< 0.001	14.9 ± 2.9	15.9 ± 4	0.34
Sino-tubular junction						
Area (cm^2^)	7 ± 1.5	7.7 ± 2.1	0.05	6 ± 1	6.2 ± 1.1	0.5
Perimeter (mm)	89.5 ± 15	97.3 ± 21.7	0.009	87.7 ± 6.8	89.2 ± 8	0.43
Diameter max (mm)	30 ± 3.4	31.4 ± 3.1	0.006	27.8 ± 2.5	28 ± 2.6	0.63
Diameter min (mm)	28.8 ± 3.1	31.3 ± 3	0.11	27.3 ± 2.4	27.7 ± 2.8	0.81
Ascending aorta						
Area (cm^2^)	9.3 ± 2.3	9.4 ± 2.6	0.24	9 ± 1.6	8.7 ± 1.4	0.48
Perimeter (mm)	108.2 ± 12.5	106.3 ± 24.3	0.25	106.9 ± 8.9	105.1 ± 8	0.45
Diameter max (mm)	34.7 ± 3.7	35.2 ± 3.8	0.3	33.6 ± 3	33.1 ± 2.7	0.5
Diameter min (mm)	34.2 ± 4	35 ± 4	0.41	33.5 ± 3.2	33 ± 3.1	0.48

Women with AS compared with women without AS got smaller sinus of Valsalva in terms of the area (7.3 ± 1 cm^2^ vs. 7.9 ± 1.4 cm^2^; p = 0.022), the perimeter (99.3 ± 6.8 mm vs. 104.6 ± 9.5 mm; p = 0.02), the noncoronary cusp diameter (30.9 ± 2.5 mm vs. 33.1. ± 3.7 mm; p = 0.018) and the distance of the aortic annulus to the STJ on the left side (18.6 ± 1.3 mm vs. 20.2 ± 1.6 mm; p = 0.001), but aortic root volume was comparable. Contrary to men, AS women compared with controls got a larger LVOT in terms of the area (4.2 ± 0.5 cm^2^ vs. 3.7 ± 0.7 mm; p = 0.005), the perimeter (77.4 ± 5.3 mm vs. 73.2 ± 6.4 mm; p = 0.015) and diameters (28.6 ± 2.5 mm vs. 27.2 ± 2.2 mm; p = 0.035; 18.9 ± 1.6 mm vs. 17.1 ± 2.5 mm; p = 0.004) and diameters of the aortic annulus (26.8 ± 1.7 mm vs. 25.1 ± 1.9 mm; p = 0.002; 20.7 ± 1.4 mm vs. 19.8 ± 1.9 mm; p = 0.05) (Table 4).

## Discussion

Our study assessed calcium score and geometry of the LVOT, the aortic root and the ascending aorta in 50 patients with AS and 50 controls who underwent computed tomography scanning due to clinical indications.

To the best of our knowledge, there is limited data for the comparison of the aortic root geometry between patients with tricuspid AS and patients without stenosis. Our study revealed that patients with tricuspid AS had smaller all dimensions of the sinus of Valsalva as well as a distance between the aortic annulus and the STJ on the right side resulting in a reduction of the aortic root volume compared with patients without stenosis. In the study using 3D transesophageal echocardiography observed reductions of aortic root size in both longitudinal and transverse directions in patients with tricuspid AS and 23% reduction of aortic root volume, but they also described smaller STJ dimensions [7]. Similar to our study, previous studies using MDCT have demonstrated that patients with AS had decreased distance from the aortic valve annulus to the STJ compared with controls, but they denied differences in cross-sectional dimensions of the aortic root [8], while other revealed a transverse remodelling of the aortic root including the diameter of the ascending aorta and the STJ without differences in longitudinal dimensions [9]. While part of previous studies are consistent with our results, there remains also controversy. Discrepancies of study results can be caused by different patient populations, different methodologies or prevalence of aortic regurgitations.

We did not observe differences in ascending aorta dimensions in patients with tricuspid AS compared with controls. The previous studies often reported that aortic stenosis may cause dilatation of the ascending aorta [10–12]. Post-stenotic aortic dilatation is defined as dilatation of the vessel wall distal to the area of a partial stenosis [12]. Extension of the aortic root occurs more frequently in patients with AS, aortic regurgitations or bicuspid aortic valve [12, 13]. The widening of the aorta is associated more frequently with bicuspid aortic valve (BAV) than tricuspid aortic stenosis (TAV) [10, 11, 13], which may results from a disorder of a wall structure in patients with BAV [11, 12].

The present study revealed an elipitic shape of the aortic annulus and the LVOT, whereas the STJ and the ascending aorta were more likely to be circular in AS patients as well as in controls. What is more, there were no differences in the Ellipticity Index in the study group compared with patients without AS, except the STJ, which was less frequently circular in the AS group. The shape of the aortic root was sex-independent. Our results are comparable to numerous studies [4, 14–16]. One study showed that in contrast to the ascending aorta, which was circular, the annulus and the LVOT were oval in shape, and the elipticity was more pronounced in the LVOT compared with the annulus [4]. Differences in the shape of the aortic root are sex-independent. In systole, the minimal diameter of the aortic annulus increased leading to a decrease in the ellipticity. The annulus assumed a more round shape, thus increasing aortic valve area (AVA) without a substantial change in perimeter [16]. These changes are slight in patients with calcified valves, because tissue properties allow very little changes [16]. The previous study emphasized that a significant oval shape of the aortic annulus may increase the risk of paravalvular leakage after TAVI [14]. The aortic annulus shape is associated with age, with more circular shape in patients younger than 40 years and oval shape in older patients [17].

The components of the aortic root have been described in previous reports but most of these studies assessed only part of the aortic root dimensions, which do not reflect geometry of the aortic root and impact of gender. Our study is one of the few assessment of anatomic dimensions at the various levels of the aortic root, which evaluated aortic root volume and showed sex differences. The age, weight and body surface area were similar in men and women, whereas height and body mass index were larger in men. The present study showed significant sex-dependent differences in the area of the LVOT, the aortic annulus and the sinus of Valsalva and the perimeter of coronary sinus in term of absolute as well as indexed to BSA. Our study showed also that diameters of the LVOT, the aortic annulus and the sinus of Valsalva and the perimeters of LVOT and sinus of Valsalva were larger in men with regard to absolute scores, but indexed values were comparable in both groups. Correlations between patients’ gender and sinus of Valsalva height was not found, but a larger sinus of Valsalva diameter was associated with male gender [18]. Significant differences between men and women were found with regard to the height of the left and right sinus of Valsalva with a larger height for men [9]. Female patients showed also smaller values of the aortic annulus and the sinus of Valsalva, when compared with men, STJ indexed to BSA remained without differences [9]. Our results are also partly consistent with study which revealed larger annulus, LVOT and sinus of Valsalva dimensions in men than in women, whereas dimensions of the ascending aorta were comparable [4].

To the best of our knowledge, there is limited data evaluating differences in aortic root geometry between tricuspid AS patients and healthy controls among women and men. Our study showed that the geometry of the aortic root in women with AS compared to healthy ones shows much more differences than an analogical comparison of men. Men differed from each other by the dimensions of the sinus of Valsalva, the STJ and aortic root volume, while comparison between women with AS and controls showed differences in the sinus of Valsalva, the LVOT, and the aortic annulus, but aortic root volume was comparable. Further research is necessary and our data may contribute to raising awareness of AS etiology.

Our results represent a single-centre study. Selection was biased towards patients with a high cardiac operation risk who were disqualified from surgical aortic replacement, not representative for the overall population of patients with severe aortic stenosis. The small sample size limited comparisons.

### Summary

MSCT successfully showed anatomic differences in aortic root geometry in AS patients compared with those without valvular pathology and indentified sex-dependent differences. Significant transverse and longitudinal aortic root remodeling were demonstrated in AS patients compared with controls in contrast to the aortic annulus, the LVOT and the STJ, which did not show differences between both groups. No significant differences in ascending aorta dimensions between tricuspid AS and controls were found. Overall, the aortic annulus and the LVOT were predominantly oval, while the ascending aorta and the STJ were circular in most patients in both groups. Male sex is associated with a greater annulus, a greater LVOT and sinus of Valsalva, but not aortic dimensions. More differences were found between tricuspid AS women and healthy women than in a similar comparison between men. Our study revealed a high variability in the aortic root dimensions between AS patients and healthy controls, which may be relevant for further research into the etiology and pathophysiology of aortic stenosis. What is more, detailed knowledge of the aortic root anatomy in patients with aortic stenosis is crucial for the further improvement of interventional treatment techniques, especially transacatheter aortic valve implantation. Recognition of the sex-dependent aortic root anatomic differences will improve the selection of appropriate systems used for TAVI in particular groups of patients, moreover, it can help identify the group of patients who will benefit most from TAVI. A high variability in the aortic root dimensions require further careful research.

## References

[1] Iung B, Baron G, Butchart EG, Delahaye F, Gohlke-Bärwolf C, Levang OW, Tornos P, Vanoverschelde JV, Vermeer F, Boersma E, Ravaud P, Vahanian A (2003). A prospective survey of patients with valvular heart disease in Europe: the Euro Heart Survey on Valvular Heart Disease. Eur Heart J.

[2] Baumgartner H, Falk V, Bax JJ, De Bonis M, Hamm C, Holm PJ, Iung B, Lancellotti P, Lansac E, Rodriguez Muñoz D, Rosenhek R, Sjögren J, Tornos Mas P, Vahanian A, Walther T, Wendler O, Windecker S, Zamorano JL (2017). 2017 ESC/EACTSGuidelines for the management of valvular heart disease. The Task Force for the Management of Valvular Heart Disease of the European Society of Cardiology (ESC) and the European Association for Cardio-Thoracic Surgery (EACTS). Eur Heart J.

[3] Piazza N, de Jaegere P, Schultz C, Becker AE, Serruys PW, Anderson RH (2008). Anatomy of the aortic valvar complex and its implications for transcatheter implantation of the aortic valve. Circulation.

[4] Buellesfeld L, Stortecky S, Kalesan B, Gloekler S, Khattab AA, Nietlispach F, Delfine V, Huber C, Eberle B, Meier B, Wenaweser P, Windecker S (2013). Aortic root dimensions among patients with severe aortic stenosis undergoing transcatheter aortic valve replacement. JACC Cardiovasc Interv.

[5] Clavel MA, Messika-Zeitoun D, Pibarot P, Aggarwal SR, Malouf J, Araoz PA, Michelena HI, Cueff C, Larose E, Capoulade R, Vahanian A, Enriquez-Sarano M (2013). The complex nature of discordant severe calcified aortic valve disease grading: new insights from combined doppler echocardiographic and computed tomographic study. J Am Coll Cardiol.

[6] Jurencak T, Turek J, Kietselaer BLJH, Mihl C, Kok M, Ommen VGVA, Garsse LAFM, Nijssen EC, Wildberger JE, Das M (2015). MDCT evaluation of aortic root and aortic valve prior to TAVI. What is the optimal imaging time point in the cardiac cycle?. Eur Radiol.

[7] Chien-Chia WuV, Kaku K, Takeuchi M, Otani K, Yoshitani H, Tamura M, Abe H, Lin FC, Otsuji Y (2014). Aortic root geometry in patients with aortic stenosis assessed by real-time three-dimensional transesophageal echocardiography. J Am Soc Echocardiogr.

[8] Akhtar M, Tuzcu EM, Kapadia SR, Svensson LG, Greenberg RK, Roselli EE, Halliburton S, Kurra V, Schoenhagen P, Sola S (2009). Aortic root morphology in patients undergoing percutaneous aortic valve replacement: evidence of aortic root remodeling. J Thorac Cardiovasc Surg.

[9] Stolzmann P, Knight J, Desbiolles L, Maier W, Scheffel H, Plass A, Kurtcuoglu V, Leschka S, Poulikakos D, Marincek B, lkadhi H (2009). Remodelling of the aortic root in severe tricuspid aortic stenosis: implications for transcatheter aortic valve implantation. Eur Radiol.

[10] Keane MG, Wiegers SE, Plappert T, Pochettino A, Bavaria JE, St MG, Sutton J (2000). Bicuspid aortic valves are associated with aortic dilatation out of proportion to coexistent valvular lesions. Circulation.

[11] Ruzmetov M, Shah JJ, Fortuna RS, Welke KF (2015). The Association between Aortic Valve Leaflet Morphology and Patterns of Aortic Dilation in Patients with Bicuspid Aortic Valves. Ann Thorac Surg.

[12] Wilton E, Jahangiri M (2006). Post-stenotic aortic dilatation. J Cardiothorac Surg.

[13] Hanis I, Linhartová K, Beránek V, Sefrna F, Hanisová I, Sterbáková G, Pesková M (2007). Aortic stenosis severity is not a risk factor for poststenotic dilatation of the ascending aorta. Circ J.

[14] Tops LF, Wood DA, Delgado V, Schuijf JD, Mayo JR, Pasupati S, Lamers FPL, van der Wall EE, Schalij MJ, Webb JG, Bax JJ (2008). Noninvasive evaluation of the aortic root with multislice computed tomography. Implications for transcatheter aortic valve replacement. JACC Cardiovasc Imaging.

[15] Jilaihawi H, Kashif M, Fontana G, Furugen A, Shiota T, Friede G, Makhija R, Doctor N, Leon MB, Makkar RR (2012). Cross-sectional computed tomographic assessment improves accuracy of aortic annular sizing for transcatheter aortic valve replacement and reduces the incidence of paravalvular aortic regurgitation. J Am Coll Cardiol.

[16] Hamdan A, Guetta V, Konen E, Goitein O, Segev A, Raanani E, Spiegelstein D, Hay I, Segni ED, Eldar M, Schwammenthal E (2012). Deformation dynamics and mechanical properties of the aortic annulus by 4-dimensional computed tomography: insights into the functional anatomy of the aortic valve complex and implications for transcatheter aortic valve therapy. J Am Coll Cardiol.

[17] Oshita C, Murata K, Wada Y, Nao T, Uchida K, Okuda S, Susa T, Tanaka N, Matsuzaki M, Yano M (2014). Assessment of the aortic valve annular geometry by real-time three-dimensional transthoracic echocardiography: comparison with two-dimensional transthoracic echocardiography and multidetector computed tomography. J Echocardiogr.

[18] Bahlmann E, Nienaber CA, Cramariuc D, Gohlke-Baerwolf C, Ray S, Devereux RB, Wachtell K, Kuck KH, Davidsen E, Gerdts E (2011). Aortic root geometry in aortic stenosis patients (a SEAS Substudy). Eur J Echocardiogr.

